# Association of changes in predicted body composition with subsequent risk of dementia

**DOI:** 10.1002/acn3.52096

**Published:** 2024-07-15

**Authors:** Sung Min Kim, Seulggie Choi, Gyeongsil Lee, Yun Hwan Oh, Joung Sik Son, Ahryoung Ko, Ji Soo Kim, Yoosun Cho, NaNa Keum, Sang Min Park

**Affiliations:** ^1^ Department of Transdisciplinary Medicine Seoul National University Hospital Seoul South Korea; ^2^ Department of Biomedical Sciences Seoul National University Graduate School Seoul South Korea; ^3^ Department of Internal Medicine Seoul National University Hospital Seoul South Korea; ^4^ Life Clinic Seoul South Korea; ^5^ KS Healthlink Institute Seoul South Korea; ^6^ Department of Family Medicine, Chung‐Ang University Gwangmyeong Hospital Chung‐Ang University College of Medicine Gwangmyeong‐si South Korea; ^7^ Department of Family Medicine Korea University Guro Hospital Seoul South Korea; ^8^ Department of Family Medicine, Seoul National University Hospital Seoul National University College of Medicine Seoul South Korea; ^9^ International Healthcare Center, Seoul National University Hospital Seoul National University College of Medicine Seoul South Korea; ^10^ Department of Food Science and Biotechnology Dongguk University Graduate School Seoul South Korea; ^11^ Department of Nutrition Harvard T.H. Chan School of Public Health Boston Massachusetts USA

## Abstract

**Objective:**

The effect of body composition change on the risk of dementia is not clear. This study analyzed the associations of changes in predicted lean body mass index (pLBMI), predicted appendicular skeletal muscle mass index (pASMI), and predicted body fat mass index (pBFMI) with the risk of dementia.

**Methods:**

In this nationwide cohort study, data were obtained from the Korean National Health Insurance Service database. The exposure was defined as changes in pLBMI, pASMI, and pBFMI derived from validated prediction equations. The outcome was dementia, defined based on the dementia diagnosis with prescription of anti‐dementia medication. Cox proportional hazards regression analyses were performed to obtain the hazard ratio with a 95% confidence interval for risk of dementia according to changes in predicted body composition.

**Results:**

A total of 13,215,208 individuals with no prior record of dementia who underwent health screenings twice between 2009–2010 and 2011–2012 were included. A 1‐kg/m^2^ increase in pLBMI and pASMI had an association with reduced risk of dementia (aHR: 0.85, 95% CI 0.84–0.87; aHR: 0.70, 95% CI 0.69–0.72, respectively for men, and aHR: 0.69, 95% CI 0.67–0.71; aHR: 0.59, 95% CI 0.57–0.61, respectively for women). A 1‐kg/m^2^ increase in pBFMI had an association with a raised risk of dementia (aHR: 1.19, 95% CI 1.17–1.21 for men and aHR: 1.53, 95% CI 1.48–1.57 for women). These results remained consistent regardless of sex or weight change.

**Interpretation:**

Increase in pLBMI or pASMI, or reduction in pBFMI was linked to lower risk of dementia.

## Introduction

Dementia is becoming more common with increased life expectancy globally. There are >55 million patients with dementia worldwide, and 10 million new cases are reported each year.^1^ Therefore, the risk factors of dementia should be investigated to reduce disease burden. Obesity is a known risk factor of cognitive deficit or dementia,^2^, ^3^, ^4^ although the relationship is inconsistent depending on different phenotypes of obesity.

Although body mass index (BMI) is an indicator of obesity, it would not wholly reflect body composition.^5^, ^6^ BMI is unable to make a distinction between lean mass and fat mass because it does not categorize body weight.^7^ Low lean mass^8^, ^9^, ^10^ and high fat mass^11^ are associated with cognitive deficit in the elderly.

The relationship between BMI‐defined obesity and risk of dementia shows a U‐shaped curve,^12^ and mid‐life obesity is a risk factor for dementia.^13^ However, being overweight or obese, as defined by BMI, has a protective effect on cognitive impairment.^14^ While some studies have suggested the association of weight loss with cognitive decline,^12^, ^15^, ^16^ one study showed that both weight gain and loss might be significant risk factors associated with dementia.^17^ These conflicting results may be attributed to the lack of consideration of body composition when using BMI as the measurement for obesity. Obesity and dementia also demonstrate a bidirectional relationship.^18^ While obesity may increase dementia risk,^13^ weight loss could lead to cognitive decline,^19^ presenting inconsistent results. Therefore, focusing solely on obesity or weight changes in dementia research is limited. Considering factors such as body composition, including fat and muscle mass, is necessary for a more accurate assessment.

Some studies have shown that patients with dementia have lower lean mass and higher fat mass than normal people,^20^, ^21^ but these studies could not reflect the temporal association of body composition with dementia because of their cross‐sectional nature.

Although direct measurements of body composition factors (dual X‐ray absorptiometry [DXA] or bioimpedance analysis) are the most accurate, their applications in primary care settings and population‐based studies are limited due to their high cost and low accessibility.^22^ Considering practical constraints within the epidemiologic environment, we utilized validated prediction equations incorporating anthropometric measurements, serum creatinine levels, and lifestyle factors impacting obesity or muscle to estimate lean body mass index (LBMI), appendicular skeletal muscle index (ASMI), and body fat mass index (BFMI).^23^ Subsequently, we explored the relationship between changes in predicted body composition and the subsequent risk of dementia.

The extensive research on differences in body composition between males and females has consistently demonstrated that males generally possess greater lean mass, while females tend to exhibit higher levels of fat mass.^24^ Additionally, various studies have indicated that vulnerability to dementia or risk factors for dementia may vary depending on sex.^25^, ^26^, ^27^ Considering these findings, our study aimed to conduct sex stratification when analyzing the association between changes in body composition and the risk of dementia.

Early‐onset dementia and late‐onset dementia share a common underlying disease process resulting in progressive cognitive decline and functional impairment. However, they also represent distinct manifestations of dementia including Alzheimer's disease (AD), differing in clinical presentation, neuropathological features, underlying causes, and social effect.^28^, ^29^, ^30^ Therefore, we aimed to analyze how changes in body composition impact the early‐onset and late‐onset dementia through age stratification.

## Methods

### Study population

Since 1989, Koreans have been receiving the benefits of national health insurance from the National Health Insurance Service (NHIS). Korean people over the age of 40 should undergo biannual health screening. Data were acquired from the NHIS retrospective cohort, which includes information about diagnosis based on the International Classification of Disease (ICD) codes (10^th^ revision), insurance claims, visiting clinical facilities, drug prescriptions, and health screening.^31^ Health screening information includes data on sociodemographic characteristics, physical examination, and health behavior of enrollees.

**Figure 1:**
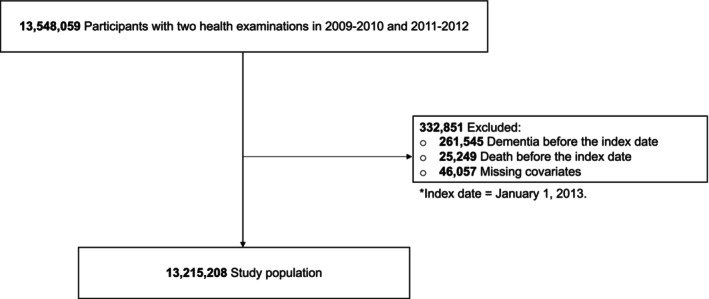
Study population flow.

The participants who were obtained from the National Health Insurance‐National Health Information Database (NHIS‐NHID) recorded from January 1, 2002, to December 31, 2020. Among the 13,548,059 adults aged 20 and older who got first and second health screenings between 2009–2010 and 2011–2012, 261,545 individuals who had dementia prior to the index date (January 1, 2013) by the ICD‐10 codes of dementia (F00, F01, F02, F03, and G30) were excluded. Furthermore, 25,249 dead participants prior to the index date and 46,057 for whom covariate data were missing were excluded. The final study population comprised 13,215,208 patients (Fig. 1).

### Exposures and outcomes

We acquired the predicted LBMI (pLBMI), ASMI (pASMI), and BFMI (pBFMI) from prediction equations (Table S1) from a previous study.^23^ Briefly, to derive prediction models, multivariable linear regressions were fitted using lean body mass (LBM), appendicular skeletal muscle mass (ASM), and body fat mass (BFM) assessed with DXA as dependent variables and predictors of body composition as independent variables (age, height, weight, waist circumference, serum creatinine levels, physical activity, alcohol consumption, and smoking status). To validate the prediction equations, the Bland–Altman plot and intraclass correlation coefficient (ICC) were applied. When implemented in a large population‐based study, the proposed prediction equations for each body composition demonstrated strong prediction performance (ICC >0.9).^23^

The exposure was changes in pLBMI, pASMI, and pBFMI collected at 2‐year intervals between the first (2009–2010) and second (2011–2012) health examinations. The changes in each index were defined by subtracting the predicted values obtained during the first health examination from those collected during the second health examination.

We set dementia as the main outcome, defined by the prescription of anti‐dementia medication with dementia diagnosis (ICD‐10 codes for F00, F01, F02, F03, or G30) from January 1, 2013, to December 31, 2020. An average follow‐up duration is 8 years. The anti‐dementia drugs included tacrine, donepezil, rivastigmine, and galantamine. The secondary outcomes were AD (F00, G30) and vascular dementia (VD) (F01). From January 1, 2013, onward, all subjects were followed up until the first occurrence of dementia outcome or mortality or December 31, 2020.

### Covariates assessment

Covariates were collected from the data of the second health screening, which was the most current period prior to the index date. They were age, sex, socioeconomic status determined by the insurance premium (%, first, second, third, or fourth quartiles), BMI (kg/m^2^, <18.5, 18.5–22.9, 23–24.9, ≥25), smoking status (never, past, and current), alcohol consumption (times per week, 0, 1–2, 3–4 and ≥5), physical activity (times per week, 0, 1–2, 3–4 and ≥5), systolic blood pressure (mmHg), fasting serum glucose level (mg/dL), total cholesterol level (mg/dL), and Charlson Comorbidity Index (0, 1, and ≥2).

### Statistical analysis

We obtained hazard ratios (HRs) and 95% confidence intervals (CIs) for dementia for every 1‐kg/m^2^ increase in each predicted body composition factor applying Cox proportional hazards model. We utilized the model adjusted for baseline for each predictor (pLBMI, pASMI, and pBFMI), baseline and secondary BMI, and the aforementioned covariates. All analyses were conducted separately by sex, and stratified analyses were performed according to baseline BMI, change in BMI from the initial (2009–2010) to the subsequent (2011–2012) health examination, and age. All individuals were followed up beginning on January 1, 2013, until the earliest occurrence of the dementia event or death or December 31, 2020.

We also acquired HRs and 95% CI of dementia per 1‐kg/m^2^ increase in pLBMI, pASMI, and pBFMI adjusted for confounders by the variation in weight status (weight stable; change in BMI ±1, weight loss; change in BMI ≤1, weight gain; change in BMI >1).

Age‐stratified analysis was conducted to determine groups that had significant associations with changes in pLBMI, pASMI, and pBFMI and dementia (age <60 and ≥60 years).

We conducted statistical analysis using SAS 9.4 software (SAS Institute, Cary, NC, USA) and considered p‐values less than 0.05 (two‐sided) as statistically significant.

### Ethical approvals

Approval for this study was obtained from the Institutional Review Board of Seoul National University Hospital (IRB number: E‐2108‐136‐1246), and since the NHIS database was constructed under strict confidentiality measures to ensure anonymization, informed consent was waived.

## Results

Among 13,215,208 individuals (7,036,653 men and 6,178,555 women), an incidence of dementia was 117,131 (1.66%) in men and 188,272 (3.04%) in women. AD and VD, respectively, developed in 97,710 (1.39%) and 2118 (0.03%) men and 166,234 (2.69%) and 2458 (0.04%) women.

Table 1 presents a summary of the characteristics of the study population. It is indicated that the participants had a mean age of 49.8 years (men: 49.2 years, women: 52.1 years). The mean BMI of men was 24.2 kg/m^2^ and women was 23.3 kg/m^2^ at the first health screening, and they increased after 2 years in both sexes. During both health screenings, men had lower pBFMI and higher pLBMI and pASMI than women.

**Table 1:** Baseline characteristics of the study population.

	Total	Men	Women
Number of people	13,215,208	7,036,653	6,178,555
Age, Mean (SD)	49.82 (13.52)	49.19 (13.43)	52.07 (13.63)
Health screening examination Period I (2009–2010)			
BMI, kg/m^2^, mean (SD)	23.77 (3.18)	24.22 (3.01)	23.26 (3.29)
pLBMI kg/m^2^, mean (SD)	16.97 (2.18)	18.44 (1.64)	15.30 (1.35)
pASMI kg/m^2^, mean (SD)	7.24 (1.20)	8.16 (0.77)	6.19 (0.59)
pBFMI kg/m^2^, mean (SD)	6.56 (1.99)	5.53 (1.41)	7.73 (1.91)
Health screening examination Period II (2011–2012)			
BMI, kg/m^2^, mean (SD)	23.85 (3.20)	24.31 (3.04)	23.31 (3.30)
pLBMI kg/m^2^, mean (SD)	17.01 (2.20)	18.50 (1.65)	15.32 (1.35)
pASMI kg/m^2^, mean (SD)	7.24 (1.22)	8.17 (0.78)	6.17 (0.57)
pBFMI kg/m^2^, mean (SD)	6.59 (2.00)	5.56 (1.42)	7.77 (1.92)
Socioeconomic status, quartile, %			
First (highest)	34.49	38.71	29.68
Second	26.85	28.99	24.42
Third	18.93	16.75	21.41
Fourth (lowest)	19.73	15.56	24.49
Physical activity, times/week, %			
0	45.12	38.70	52.43
1–2	19.90	22.32	17.14
3–4	14.84	16.63	12.79
≥5	20.15	22.35	17.64
Smoking status, %			
Never	61.27	30.80	95.97
Former	16.36	29.42	1.50
Current	22.37	39.79	2.54
Alcohol consumption, times per week, %			
0	52.97	32.35	76.46
1–2	33.69	45.71	20.00
3–4	9.58	15.59	2.73
≥5	3.76	6.35	0.81
Systolic blood pressure, mmHg, mean (SD)	122.40 (14.83)	124.61 (13.86)	119.90 (15.48)
Fasting serum glucose, mg/dL, mean (SD)	98.14 (22.90)	100.32 (24.87)	95.65 (20.13)
Total cholesterol, mg/dL, mean (SD)	195.75 (37.14)	194.66 (36.73)	196.99 (37.58)
Charlson comorbidity index, %			
0	52.31	56.27	47.80
1	25.20	23.77	26.81
≥2	22.49	19.96	25.38

BMI, body mass index; pASMI, predicted appendicular skeletal muscle mass index; pBFMI, predicted body fat mass index; pLBMI, predicted lean body mass index; SD, standard deviation.

Table 2 shows associations between changes in pLBMI, pASMI, and pBFMI and the risk of dementia. Our findings suggest a significant association between an increase of 1‐kg/m^2^ in each pLBMI and pASMI and a reduced risk of overall dementia. In men, the adjusted hazard ratio (aHR) was 0.85 (95% CI 0.84–0.87) for pLBMI and 0.70 (95% CI 0.69–0.72) for pASMI. In women, the aHR was 0.69 (95% CI 0.84–0.71) for pLBMI and 0.59 (95% CI 0.57–0.61) for pASMI. However, an increase in pBFMI of 1‐kg/m^2^ had an association with an elevated risk of overall dementia in men (aHR: 1.19, 95% CI 1.17–1.21) and women (aHR: 1.53, 95% CI 1.48–1.57). An increase of 1‐kg/m^2^ in both pLBMI and pASMI had significant association with a reduced AD risk. In men, the aHR was 0.86 (95% CI 0.84–0.87) for pLBMI and 0.71 (95% CI 0.69–0.73) for pASMI. In women, the aHR was 0.68 (95% CI 0.66–0.70) for pLBMI and 0.58 (95% CI 0.56–0.60) for pASMI. However, an increase in pBFMI of 1‐kg/m^2^ had an association with an elevated risk of AD in men (aHR: 1.18, 95% CI 1.16–1.20) and women (aHR: 1.54, 95% CI 1.49–1.58). An increase in each pLBMI and pASMI of 1‐kg/m^2^ had an association with a decrease in the risk of VD among women (aHR: 0.67, 95% CI 0.52–0.86; aHR: 0.67, 95% CI 0.51–0.87, respectively), while there was an association between a 1‐kg/m^2^ increase in pBFMI and an elevated VD risk among women (aHR: 1.59, 95% CI 1.22–2.06). Among men, the association of each 1‐kg/m^2^ rise in pLBMI, pASMI, and pBFMI with the risk of VD showed similar trends with the risk of overall dementia and AD, but without statistical significance. For baseline weight‐stratified analysis, similar trends to the primary findings were observed when examining the effect of raising each predicted value on the risk of dementia, except in men with normal weight at baseline.

**Table 2:** Hazard ratios (95% CI) of dementia per 1‐kg/m^2^ increase in change in pLBMI, pASMI, and pBFMI stratified by the baseline weight status.

Baseline weight status	Events (*n*)	Person‐year	aHR (95% CI)		
			pLBMI	pASMI	pBFMI
Men					
Overall dementia					
Overall	117,131	54,481,283	0.85 (0.84–0.87)***	0.70 (0.69–0.72)***	1.19 (1.17–1.21)***
Normal (BMI 18.5–22.9)	49,536	18,512,079	0.91 (0.88–0.93)***	0.76 (0.73–0.80)***	1.11 (1.08–1.14)***
Overweight (BMI 23–24.9)	31,970	15,008,610	0.85 (0.82–0.88)***	0.70 (0.66–0.74)***	1.19 (1.15–1.23)***
Obese (BMI ≥25)	35,625	20,960,594	0.81 (0.79–0.83)***	0.67 (0.64–0.71)***	1.25 (1.21–1.29)***
Alzheimer's disease					
Overall	97,710	54,746,769	0.86 (0.84–0.87)***	0.71 (0.69–0.73)***	1.18 (1.16–1.20)***
Normal (BMI 18.5–22.9)	42,161	18,609,390	0.92 (0.89–0.94)***	0.77 (0.73–0.81)***	1.10 (1.07–1.14)***
Overweight (BMI 23–24.9)	26,485	15,083,815	0.85 (0.82–0.89)***	0.70 (0.66–0.75)***	1.18 (1.14–1.23)***
Obese (BMI ≥25)	29,064	21,053,565	0.82 (0.79–0.84)***	0.68 (0.64–0.72)***	1.24 (1.20–1.28)***
Vascular dementia					
Overall	2118	55,102,064	0.95 (0.84–1.09)	0.83 (0.67–1.03)	1.06 (0.93–1.21)
Normal (BMI 18.5–22.9)	822	18,760,739	1.07 (0.86–1.33)	1.03 (0.72–1.46)	0.95 (0.77–1.18)
Overweight (BMI 23–24.9)	585	15,181,223	0.87 (0.67–1.11)	0.75 (0.49–1.13)	1.16 (0.90–1.49)
Obese (BMI ≥25)	711	21,160,102	0.94 (0.75–1.17)	0.75 (0.52–1.07)	1.10 (0.88–1.37)
Women					
Overall dementia					
Overall	188,272	47,757,731	0.69 (0.67–0.71)***	0.59 (0.57–0.61)***	1.53 (1.48–1.57)***
Normal (BMI 18.5–22.9)	67,862	24,103,753	0.69 (0.66–0.73)***	0.59 (0.56–0.63)***	1.50 (1.43–1.58)***
Overweight (BMI 23–24.9)	47,377	10,643,850	0.66 (0.63–0.70)***	0.56 (0.52–0.60)***	1.58 (1.49–1.68)***
Obese (BMI ≥25)	73,033	13,010,128	0.69 (0.66–0.72)***	0.62 (0.59–0.65)***	1.52 (1.45–1.60)***
Alzheimer's disease					
Overall	166,234	48,158,969	0.68 (0.66–0.70)***	0.58 (0.56–0.60)***	1.54 (1.49–1.58)***
Normal (BMI 18.5–22.9)	60,549	24,240,379	0.69 (0.66–0.73)***	0.58 (0.55–0.61)***	1.51 (1.43–1.60)***
Overweight (BMI 23–24.9)	41,887	10,748,400	0.65 (0.61–0.69)***	0.54 (0.50–0.58)***	1.61 (1.51–1.71)***
Obese (BMI ≥25)	63,798	13,170,190	0.69 (0.66–0.73)***	0.61 (0.58–0.64)***	1.52 (1.45–1.60)***
Vascular dementia					
Overall	2458	48,854,940	0.67 (0.52–0.86)**	0.67 (0.51–0.87)**	1.59 (1.22–2.06)***
Normal (BMI 18.5–22.9)	852	24,494,144	0.59 (0.38–0.93)*	0.60 (0.37–0.98)*	1.90 (1.19–3.04)**
Overweight (BMI 23–24.9)	665	10,925,497	0.50 (0.31–0.82)**	0.38 (0.22–0.66)***	2.05 (1.22–3.44)***
Obese (BMI ≥25)	941	13,435,298	0.80 (0.55–1.17)	0.89 (0.60–1.33)	1.27 (0.84–1.92)

aHR (95% CI) were calculated by Cox hazards regression analysis after adjusting for baseline each predictor, age, socioeconomic status, baseline and secondary BMI, physical activity, smoking status, alcohol consumption, systolic blood pressure, fasting serum glucose, total cholesterol, and Charlson comorbidity index.

aHR, Adjusted hazard ratio; BMI, Body mass index; CI, confidence interval; n, the number of people; pASMI, predicted appendicular skeletal muscle mass index; pBFMI, predicted body fat mass index; pLBMI, predicted lean body mass index.

* *p* < 0.05;

** *p* < 0.01;

*** *p* < 0.001.

Table 3 presents associations between changes in pLBMI, pASMI, and pBFMI and the risk of dementia according to changes in weight status. Individuals who maintained a consistent weight (change in BMI ± 1 kg/m^2^) had a lower overall dementia risk for each 1‐kg/m^2^ increase in pLBMI and pASMI, in both men (aHR: 0.86, 95% CI 0.84–0.88; aHR: 0.71, 95% CI 0.68–0.73) and women (aHR: 0.68, 95% CI 0.65–0.70; aHR: 0.58, 95% CI 0.55–0.60), respectively. However, they showed an elevated risk of overall dementia for every 1‐kg/m^2^ increase in pBFMI in men (aHR: 1.18, 95% CI 1.16–1.21) and women (aHR: 1.55, 95% CI 1.49–1.62). Individuals who experienced weight loss (change in BMI <‐1 kg/m^2^) had a decreased risk of overall dementia for each 1‐kg/m^2^ increase in pLBMI (men aHR: 0.86, 95% CI 0.83–0.89; women aHR: 0.77, 95% CI 0.73–0.82) and pASMI (men aHR: 0.73, 95% CI 0.69–0.77; women aHR: 0.68, 95% CI 0.64–0.73). Conversely, they showed an elevated risk of overall dementia for every 1‐kg/m^2^ increase in pBFMI (men aHR: 1.18, 95% CI 1.13–1.22; women aHR: 1.35, 95% CI 1.27–1.43). Individuals who experienced weight gain (change in BMI >1 kg/m^2^) showed a decreased risk of overall dementia for every 1‐kg/m^2^ increase in pLBMI (men aHR: 0.83, 95% CI 0.80–0.87; women aHR: 0.64, 95% CI 0.60–0.68) and pASMI (men aHR: 0.68, 95% CI 0.64–0.73; women aHR: 0.58, 95% CI 0.54–0.62). Conversely, they showed an elevated risk of overall dementia for each 1‐kg/m^2^ increase in pBFMI (men aHR: 1.21, 95% CI 1.16–1.21; women aHR: 1.64, 95% CI 1.54–1.75). Associations between each 1‐kg/m^2^ increase in pLBMI, pASMI, and pBFMI and AD and VD risk stratified by weight status change showed similar trends with the overall risk of dementia, but these trends were more evident in AD than in VD in both sexes.

**Table 3:** Hazard ratios (95% CI) of dementia per 1‐kg/m^2^ increase in change in pLBMI, pASMI, and pBFMI by the change in weight status.

Category	Events (*n*)	Person‐year	aHR (95% CI)		
			pLBMI	pASMI	pBFMI
Men					
Overall dementia					
Weight stable (change in BMI ± 1)	72,939	36,191,350	0.86 (0.84–0.88)***	0.71 (0.68–0.73)***	1.18 (1.16–1.21)***
Weight loss (change in BMI ≤‐1)	23,704	7,083,631	0.86 (0.83–0.89)***	0.73 (0.69–0.77)***	1.18 (1.13–1.22)***
Weight gain (change in BMI >1)	20,488	11,206,302	0.83 (0.80–0.87)***	0.68 (0.64–0.73)***	1.21 (1.16–1.26)***
Alzheimer's disease					
Weight stable (change in BMI ± 1)	60,602	36,360,832	0.86 (0.84–0.89)***	0.72 (0.69–0.75)***	1.17 (1.14–1.20)***
Weight loss (change in BMI ≤‐1)	20,038	7,132,711	0.86 (0.82–0.89)***	0.73 (0.68–0.77)***	1.18 (1.13–1.22)***
Weight gain (change in BMI >1)	17,070	11,253,226	0.84 (0.81–0.87)***	0.69 (0.64–0.74)***	1.20 (1.15–1.25)***
Vascular dementia					
Weight stable (change in BMI ± 1)	1310	36,580,925	0.95 (0.80–1.13)	0.85 (0.64–1.12)	1.08 (0.91–1.28)
Weight loss (change in BMI ≤‐1)	436	7,205,283	1.00 (0.76–1.31)	0.88 (0.56–1.36)	0.99 (0.76–1.30)
Weight gain (change in BMI >1)	372	11,315,856	0.89 (0.67–1.18)	0.69 (0.43–1.11)	1.16 (0.87–1.54)
Women					
Overall dementia					
Weight stable (change in BMI ± 1)	108,678	31,014,240	0.68 (0.65–0.70)***	0.58 (0.55–0.60)***	1.55 (1.49–1.62)***
Weight loss (change in BMI ≤‐1)	45,091	7,294,884	0.77 (0.73–0.82)***	0.68 (0.64–0.73)***	1.35 (1.27–1.43)***
Weight gain (change in BMI >1)	34,503	9,448,607	0.64 (0.60–0.68)***	0.58 (0.54–0.62)***	1.64 (1.54–1.75)***
Alzheimer's disease					
Weight stable (change in BMI ± 1)	95,576	31,256,849	0.67 (0.64–0.70)***	0.56 (0.54–0.59)***	1.57 (1.50–1.64)***
Weight loss (change in BMI ≤‐1)	40,299	7,379,053	0.78 (0.73–0.82)***	0.68 (0.64–0.73)***	1.34 (1.26–1.42)***
Weight gain (change in BMI >1)	30,359	9,523,067	0.63 (0.59–0.68)***	0.55 (0.52–0.60)***	1.66 (1.55–1.78)***
Vascular dementia					
Weight stable (change in BMI ± 1)	1433	31,657,306	0.57 (0.41–0.80)**	0.56 (0.39–0.81)**	1.89 (1.32–2.70)***
Weight loss (change in BMI ≤‐1)	593	7,546,133	1.11 (0.73–1.68)	1.13 (0.77–1.65)	0.97 (0.59–1.59)
Weight gain (change in BMI >1)	432	9,651,500	0.53 (0.30–0.93)*	0.53 (0.28–1.00)	1.83 (1.01–3.31)*

aHR (95% CI) were calculated by Cox hazards regression analysis after adjusting for baseline each predictor, age, socioeconomic status, baseline and secondary BMI, physical activity, smoking status, alcohol intake, systolic blood pressure, fasting serum glucose, total cholesterol, and Charlson comorbidity index.

aHR, Adjusted hazard ratio; BMI, Body mass index; CI, confidence interval; n, the number of people; pASMI, predicted appendicular skeletal muscle mass index; pBFMI, predicted body fat mass index; pLBMI, predicted lean body mass index.

* *p* < 0.05;

** *p* < 0.01;

*** *p* < 0.001.

Table 4 shows associations between changes in pLBMI, pASMI, and pBFMI and overall dementia risk stratified by age (<60 and ≥60 years) and baseline BMI. Regardless of age stratification, the association of each 1‐kg/m^2^ increase in pLBMI, pASMI, and pBFMI with dementia risk by the change in weight status showed similar trends as the main analysis (Table 2), but these trends were more evident in individuals with AD than in those with VD among men and women.

**Table 4:** Hazard ratios (95% CI) of dementia per 1‐kg/m^2^ increase in change in pLBMI, pASMI, and pBFMI stratified by the baseline weight status and age.

Baseline weight status	Events (*n*)	Person‐year	aHR (95% CI)		
			LBMI	ASMI	BFMI
Age <60					
Men					
Overall dementia					
Overall	7754	42,723,890	0.78 (0.73–0.84)***	0.56 (0.49–0.63)***	1.30 (1.21–1.39)***
Normal (BMI 18.5–22.9)	2865	14,359,696	0.85 (0.75–0.96)**	0.59 (0.48–0.74)***	1.20 (1.06–1.36)**
Overweight (BMI 23–24.9)	2068	11,606,071	0.81 (0.70–0.93)**	0.56 (0.43–0.72)***	1.27 (1.10–1.46)***
Obese (BMI ≥25)	2821	16,758,123	0.72 (0.65–0.80)***	0.54 (0.44–0.66)***	1.39 (1.25–1.55)***
Alzheimer's disease					
Overall	4946	42,770,517	0.80 (0.73–0.87)***	0.57 (0.49–0.67)***	1.26 (1.16–1.38)***
Normal (BMI 18.5–22.9)	1891	14,374,941	0.89 (0.76–1.04)	0.64 (0.49–0.84)**	1.14 (0.98–1.33)
Overweight (BMI 23–24.9)	1273	11,618,981	0.81 (0.67–0.97)*	0.56 (0.40–0.77)***	1.26 (1.06–1.51)*
Obese (BMI ≥25)	1782	16,776,595	0.73 (0.64–0.84)***	0.54 (0.42–0.70)***	1.37 (1.20–1.57)***
Vascular dementia					
Overall	224	42,791,777	1.11 (0.71–1.74)	1.01 (0.48–2.14)	0.92 (0.59–1.45)
Normal (BMI 18.5–22.9)	83	14,383,010	1.41 (0.66–3.00)	1.83 (0.51–6.56)	0.72 (0.34–1.54)
Overweight (BMI 23–24.9)	52	11,624,662	0.83 (0.33–2.10)	0.62 (0.12–3.06)	1.25 (0.50–3.11)
Obese (BMI ≥25)	89	16,784,105	1.08 (0.54–2.18)	0.83 (0.26–2.62)	0.93 (0.46–1.87)
Women					
Overall dementia					
Overall	9882	34,328,537	0.51 (0.44–0.59)***	0.37 (0.31–0.44)***	2.16 (1.84–2.53)***
Normal (BMI 18.5–22.9)	3927	19,812,157	0.55 (0.43–0.72)***	0.35 (0.26–0.48)***	2.01 (1.53–2.64)***
Overweight (BMI 23–24.9)	2476	7,029,847	0.49 (0.36–0.67)***	0.42 (0.29–0.61)***	2.20 (1.59–3.04)***
Obese (BMI ≥25)	3479	7,486,533	0.47 (0.37–0.59)***	0.36 (0.27–0.48)***	2.32 (1.81–2.98)***
Alzheimer's disease					
Overall	7168	34,398,158	0.48 (0.41–0.58)***	0.32 (0.26–0.40)***	2.25 (1.87–2.71)***
Normal (BMI 18.5–22.9)	2861	19,841,079	0.53 (0.39–0.72)***	0.32 (0.22–0.47)***	2.14 (1.56–2.94)***
Overweight (BMI 23–24.9)	1792	7,047,793	0.47 (0.33–0.67)***	0.35 (0.23–0.54)***	2.24 (1.54–3.28)***
Obese (BMI ≥25)	2515	7,509,285	0.44 (0.34–0.58)***	0.31 (0.22–0.43)***	2.43 (1.82–3.25)***
Vascular dementia					
Overall	164	34,438,187	0.46 (0.14–1.45)	0.40 (0.11–1.49)	2.45 (0.72–8.37)
Normal (BMI 18.5–22.9)	73	19,857,492	0.14 (0.02–0.98)*	0.22 (0.02–2.15)	6.47 (0.85–48.98)
Overweight (BMI 23–24.9)	37	7,058,091	1.14 (0.15–9.03)	0.77 (0.07–8.15)	0.92 (0.08–10.78)
Obese (BMI ≥25)	54	7,522,604	0.52 (0.08–3.40)	0.37 (0.04–3.45)	2.59 (0.36–18.76)
Age ≥60					
Men					
Overall dementia					
Overall	109,377	11,757,393	0.86 (0.85–0.88)***	0.72 (0.70–0.74)***	1.17 (1.15–1.19)***
Normal (BMI 18.5–22.9)	46,671	4,152,383	0.91 (0.89–0.94)***	0.77 (0.74–0.81)***	1.11 (1.07–1.14)***
Overweight (BMI 23–24.9)	29,902	3,402,539	0.85 (0.83–0.88)***	0.71 (0.67–0.75)***	1.18 (1.14–1.22)***
Obese (BMI ≥25)	32,804	4,202,471	0.82 (0.79–0.84)***	0.68 (0.65–0.72)***	1.24 (1.20–1.28)***
Alzheimer's disease					
Overall	92,764	11,976,252	0.87 (0.85–0.88)***	0.72 (0.70–0.74)***	1.17 (1.15–1.19)***
Normal (BMI 18.5–22.9)	40,270	4,234,448	0.92 (0.89–0.95)***	0.78 (0.74–0.81)***	1.10 (1.07–1.13)***
Overweight (BMI 23–24.9)	25,212	3,464,834	0.86 (0.83–0.89)***	0.71 (0.67–0.76)***	1.18 (1.13–1.22)***
Obese (BMI ≥25)	27,282	4,276,970	0.82 (0.80–085)***	0.69 (0.65–0.73)***	1.23 (1.19–1.27)***
Vascular dementia					
Overall	1894	12,310,288	0.96 (0.84–1.10)	0.83 (0.66–1.03)	1.07 (0.93–1.22)
Normal (BMI 18.5–22.9)	739	4,377,729	1.05 (0.84–1.32)	0.98 (0.68–1.42)	0.97 (0.77–1.22)
Overweight (BMI 23–24.9)	533	3,556,562	0.87 (0.67–1.13)	0.76 (0.49–1.17)	1.15 (0.89–1.50)
Obese (BMI ≥25)	622	4,375,997	0.93 (0.73–1.17)	0.74 (0.50–1.08)	1.12 (0.89–1.41)
Women					
Overall dementia					
Overall	178,390	13,429,194	0.70 (0.68–0.72)***	0.59 (0.57–0.61)***	1.52 (1.47–1.56)***
Normal (BMI 18.5–22.9)	63,935	4,291,596	0.70 (0.66–0.73)***	0.58 (0.54–0.61)***	1.51 (1.43–1.59)***
Overweight (BMI 23–24.9)	44,901	3,614,003	0.67 (0.64–0.72)***	0.56 (0.52–0.59)***	1.56 (1.47–1.66)***
Obese (BMI ≥25)	69,554	5,523,595	0.70 (0.67–0.74)***	0.62 (0.59–0.66)***	1.50 (1.43–1.57)***
Alzheimer's disease					
Overall	159,066	13,760,811	0.69 (0.67–0.71)***	0.58 (0.56–0.60)***	1.53 (1.48–1.58)***
Normal (BMI 18.5–22.9)	57,688	4,399,300	0.69 (0.66–0.73)***	0.56 (0.53–0.60)***	1.52 (1.43–1.60)***
Overweight (BMI 23–24.9)	40,095	3,700,607	0.66 (0.62–0.70)***	0.54 (0.50–0.57)***	1.59 (1.49–1.70)***
Obese (BMI ≥25)	61,283	5,660,905	0.70 (0.67–0.74)***	0.62 (0.58–0.65)***	1.50 (1.43–1.58)***
Vascular dementia					
Overall	2294	14,416,753	0.68 (0.53–0.87)**	0.66 (0.50–0.87)**	1.58 (1.21–2.07)***
Normal (BMI 18.5–22.9)	779	4,636,653	0.63 (0.40–1.00)	0.58 (0.35–0.97)*	1.85 (1.13–3.01)*
Overweight (BMI 23–24.9)	628	3,867,406	0.49 (0.29–0.81)**	0.36 (0.20–0.64)***	2.12 (1.24–3.61)**
Obese (BMI ≥25)	887	5,912,694	0.82 (0.56–1.21)	0.92 (0.61–1.37)	1.23 (0.81–1.88)

aHR (95% CI) were calculated by Cox hazards regression analysis after adjusting for baseline each predictor, age, socioeconomic status, baseline and secondary BMI, physical activity, smoking status, alcohol intake, systolic blood pressure, fasting serum glucose, total cholesterol, and Charlson comorbidity index.

aHR, Adjusted hazard ratio; BMI, Body mass index; CI, confidence interval; n, the number of people; pASMI, predicted appendicular skeletal muscle mass index; pBFMI, predicted body fat mass index; pLBMI, predicted lean body mass index.

* *p* < 0.05;

** *p* < 0.01;

*** *p* < 0.001.

## Discussion

We investigated whether changes in pLBMI, pBFMI, and pASMI influence the risk of overall dementia over an average of 8 years of follow‐up. Increased pLBMI and pASMI or decreased pBFMI were related to a decreased risk of dementia. Those trends were consistent regardless of weight change in both sexes.

The association between body composition, skeletal muscle or fat mass, and dementia has been established. High adiposity related to increased fat mass is correlated with brain atrophy^32^, ^33^, ^34^ and systemic inflammation, which can precipitate neurodegenerative changes.^35^, ^36^, ^37^ Obesity could increase the risk of dementia by atherosclerosis, endothelial dysfunction, and defects in the blood–brain barrier (BBB).^38^, ^39^ Although the basis for association between muscle mass and dementia is unclear,^40^ inflammation caused by aging might contribute to skeletal muscle alterations, sarcopenia, and AD.^41^ In addition, decreased physical function induces endothelial dysfunction, which can cause cognitive impairment.^42^

There were some studies of the association of changes in BMI with the risk of dementia. High BMI is associated with a raised dementia risk^43^; however, high BMI in old age provides a protective effect against the risk of dementia.^43^, ^44^, ^45^, ^46^ This inverse association could be explained by leptin from adipose tissue. Elevated leptin levels can control hippocampal synaptic plasticity and processing of amyloid beta, leading to a reduced dementia risk.^3^ Compared with the younger population, a different pattern of weight loss was observed among the elderly when assessing dementia risk. Elderly individuals with a low BMI or underweight or weight loss are at higher risk of dementia compared with normal or stable BMI.^43^ This could be explained by some physiological mechanisms. Because reduced calorie intake causes weight loss, subsequent macronutrient and micronutrient deficiencies may exacerbate cognitive performance.^47^ In the elderly, bone loss and sarcopenia are important to weight loss. Decreased bone mass may elevate inflammatory markers related to the risk of dementia.^47^ Therefore, regular weight training may have preventive effect on dementia risk of old ages. Moreover, prior research has demonstrated a correlation of increased body weight variability with elevated dementia risk in older adults.^48^ These results could be explained by body weight fluctuations related to poor body fat distribution.^49^ Body weight fluctuations may indicate difficulty in maintaining homeostasis and subsequent cognitive impairment.^48^

In our study, we found that an increase in pLBMI and pASMI was linked to a lower risk of dementia, while an increase in pBFMI was associated with a higher risk of dementia, and this pattern held consistent across individuals with any baseline BMI or weight stable, loss, or gain. Our study implicates that consideration of changes in muscle mass and fat mass might be a more effective indicator for estimating the risk of dementia compared to only considering body weight and its changes. In particular, we suggest that even if weight loss or gain outside the normal BMI category in elderly people have a positive association with the risk of dementia, the risk could be attenuated by adjusting detailed body composition factors such as increasing muscle mass and reducing BFM. Generally, weight gain is caused by increased fat mass; however, weight loss is caused by reduced fat and muscle mass. When we distinguished each body composition factor from composite body weight, we identified that decreased BFM had an association with a reduced risk of dementia, but decreased LBM or ASM were related to higher risk of dementia. These results implicated that the association of weight loss with the dementia risk might be caused by muscle loss rather than fat, which indicates that weight loss alone may not always be effective in preventing dementia.

Associations of body composition change with the risk of both AD and VD showed similar trends in our study. Managing body weight could be important to prevent dementia as diseases related to body weight, particularly obesity, could increase the risk of cardiovascular disease^50^ and induce hypertension and insulin resistance,^51^, ^52^ which are related to the risk of AD and VD.

When we analyzed participants based on age, dividing them into under 60 and 60 and older, we observed that those under 60 years were generally more sensitive to the risk of dementia linked with changes in body composition compared to those aged 60 and above. The elderly population aged 60 and above, being at a high risk for dementia, may experience a weakened modifiable effect on dementia risk due to changes in muscle mass and fat mass compared to younger age groups. Moreover, our results may suggest the presence of a “legacy effect,” indicating that managing modifiable factors from a younger age could be effective in preventing diseases and maintaining health later in life. Several studies emphasize the importance of interventions from early age, such as controlling modifiable factors for dementia prevention,^53^ and even suggest the necessity of dementia prevention measures from birth.^54^ Therefore, based on our findings, it is estimated that increasing muscle mass and reducing fat mass from the early stages may be more effective in preventing dementia that could occur later on.

This study discovered a stronger association between changes in body composition and dementia risk in women compared to men, which might mean that women not only have a higher susceptibility to dementia but also exhibit greater vulnerability to changes in body composition in relation to dementia risk compared to men. Furthermore, the risk of dementia is higher in women compared to men,^55^, ^56^ which is primarily because women have greater longevity as the risk of dementia increases with age.^57^ In addition, this phenomenon might be linked to differences in body composition between the sexes. Compared to men, women have less muscle but more fat,^58^ and this difference might be related to high sensitivity to the impact of change in body composition on the risk of dementia. In a previous study, BBB disruption by obesity was increased in overweight and obese women only.^59^ However, the sex differences in VD are unclear. A previous study reported that the incidence of VD is greater in women than men,^60^ but the opposite result has also been reported.^55^ As no clear mechanism has yet been elucidated on sex differences in dementia risk, more detailed mechanism studies are needed. Also, in our study, in men, the benefits of increased LBMI and ASMI and risks of increased BFMI in terms of risk of dementia were similar regardless of weight change in men. However, in women, the least benefits of increased LBMI and ASMI were seen in the weight loss group while the highest risk from increased BFMI was seen in the weight gain group. The findings may suggest that weight changes interact differently with changes in muscle or fat mass and the risk of dementia between women and men. Women typically have a higher likelihood of developing metabolic syndrome compared to men.^61^ Metabolic syndrome is linked to an increased risk of dementia,^62^ and as fat mass rise, so does the risk of metabolic syndrome.^63^ Therefore, an increase in body weight and fat mass could potentially elevate the risk of conditions like metabolic syndrome, consequently increasing the risk of dementia. However, the reason for the relatively low preventive effect of muscle mass increase on dementia in women with weight loss compared to other groups remains unclear. Women's weight loss and muscle mass changes are influenced by various physiological, behavioral, and social factors,^64^, ^65^ but the underlying mechanisms of how these factors relate to the dementia have not yet been clarified. Further research is needed to clarify this relationship.

This study has some limitations. Firstly, while the prediction equations we used have sufficient predictive power,^23^ it may be beneficial to obtain longitudinal muscle mass and fat mass information from participants' whole‐body DXA as additional validation to enhance their reliability. Secondly, NHIS cohort data does not include some confounding factors such as homeostatic model assessment for insulin resistance (HOMA‐IR), indicating insulin resistance, total calorie intake, or muscle strength.

This study also has strengths. Firstly, we found the association between changes in each factor of body composition and the subsequent risk of dementia for the first time. Secondly, for a large‐scale, nationwide epidemiological study, changes in each body composition factor were assessed by validated anthropometric measures without using complex measurement devices. Lastly, to the best of our knowledge, this appears to be the first study focusing on assessing the combined association between changes in weight and body composition on the risk of dementia.

In conclusion, a decrease in pLBMI and pASMI or increase in pBFMI indicated a higher dementia risk, but an increase in pLBMI and pASMI or a decrease in pBFMI had a protective effect on the risk of dementia. This pattern is consistent across individuals with any baseline BMI or weight stable, loss, or gain. Intentionally increasing muscle mass and reducing fat mass may help lower the risk of dementia in any age group.

## Conflict of Interest

None of the authors reported disclosures.

## Author Contributions

S.M. Park had full access to all of the data in the study, takes responsibility for the integrity of the data and the accuracy of the data analysis, and involved in administrative, technical, or material support. S.M. Kim involved in study concept and design, drafting of the manuscript, statistical analysis, and acquisition of data. All authors involved in analysis and interpretation of data, and critical revision of the manuscript.

## Supporting information


Table S1.

## Data Availability

The database utilized in this study is the property of the NHIS, and the authors are not authorized to disclose the data employed in this study.
