# Supplementary material for: Association of changes in predicted body composition with subsequent risk of dementia
Source: Ann Clin Transl Neurol. 2024 Jul 15;11(8):1952–63. doi: 10.1002/acn3.52096 (PMC11330214; doi:10.1002/acn3.52096)
Supplement: Supplementary file 1 — Table S1. [file ACN3-11-1952-s001.docx]

**Supplementary Table 1.** Anthropometric prediction equations for lean body mass index, appendicular skeletal muscle mass index, and body fat mass index

| Men | LBMI | -0.296 + 0.012*age (years) + 0.134*height (cm) + 0.675*weight (kg) - 0.201*waist (cm) - 0.249*serum creatinine (mg/dL) + 0.270*moderate physical activity + 0.924*vigorous physical activity – 0.559*past smoker + 0.234*current smoker - 0.046*moderate drinker + 0.324*heavy drinker |
| --- | --- | --- |
|  | ASMI | -2.236 - 0.011*age (years) + 0.081*height (cm) + 0.324*weight (kg) - 0.121*waist (cm) – 0.008*serum creatinine (mg/dL) + 0.200*moderate physical activity + 0.587*vigorous physical activity – 0.195*past smoker – 0.016*current smoker + 0.004*moderate drinker + 0.151*heavy drinker |
|  | BFMI | 0.561 - 0.012*age (years) - 0.133*height (cm) + 0.310*weight (kg) + 0.199*waist (cm) + 0.253*serum creatinine (mg/dL) - 0.247*moderate physical activity - 0.878*vigorous physical activity + 0.708*past smoker - 0.275*current smoker + 0.077*moderate drinker - 0.291*heavy drinker |
| Women | LBMI | -11.941 + 0.015*age (years) + 0.171*height (cm) + 0.457*weight (kg) - 0.060*waist (cm) + 0.428*serum creatinine (mg/dL) + 0.181*moderate physical activity + 0.654*vigorous physical activity – 0.254*past smoker + 0.221*current smoker + 0.209*moderate drinker + 0.126*heavy drinker |
|  | ASMI | -8.447 + 0.002*age (years) + 0.091*height (cm) + 0.203*weight (kg) - 0.034*waist (cm) + 0.539*serum creatinine (mg/dL) + 0.103*moderate physical activity + 0.362*vigorous physical activity – 0.203*past smoker + 0.000*current smoker + 0.103*moderate drinker + 0.031*heavy drinker |
|  | BFMI | 12.269 - 0.014*age (years) - 0.172*height (cm) + 0.530*weight (kg) + 0.058*waist (cm) – 0.314*serum creatinine (mg/dL) - 0.123*moderate physical activity - 0.541*vigorous physical activity + 0.261*past smoker - 0.242*current smoker - 0.218*moderate drinker - 0.182*heavy drinker |

LBMI Lean body mass index; ASM Appendicular skeletal muscle mass index; BFM Body fat mass index; MET Metabolic Equivalent Task.

The prediction equations were developed and validated based on data from the Korean National Health and Nutrition Examination Survey 2008–2011.

Moderate physical activity: those who engage in 600-2999 METs-min/week of physical activity.

Vigorous physical activity: those who engage in ≥3000 METs-min/week of physical activity.

Moderate drinker: drinking alcohol less than 14 drinks per week (men), or less than 7 drinks per week (women).

Heavy drinker: drinking alcohol 14 drinks or more per week (men), or 7 drinks or more per week (women).

The variables of physical activity, smoking, and alcohol consumption were represented as binary values (1 for yes, 0 for no).
